# Genetically determined ABO blood group, secretor status, Lewis antigens, and panNEN risk

**DOI:** 10.1530/ERC-26-0286

**Published:** 2026-09-18

**Authors:** Samuel O Antwi, Erin E Carlson, Kari G Rabe, Teja Koganti, William R Bamlet, MAYO-RGC Project Generation, Regeneron Genetics Center, Thorvardur R Halfdanarson, Robert R McWilliams, Kirk J Wangensteen, Ann L Oberg

**Affiliations:** ^1^ Division of Epidemiology, Department of Quantitative Health Sciences, Mayo Clinic, Jacksonville, Florida, USA; ^2^ Department of Quantitative Health Sciences, Mayo Clinic, Rochester, Minnesota, USA; ^3^ Department of Medical Oncology, Mayo Clinic, Rochester, Minnesota, USA; ^4^ OSF HealthCare Cancer Institute, University of Illinois, Peoria, Illinois, USA; ^5^ Division of Gastroenterology and Hepatology, Department of Medicine, Mayo Clinic, Rochester, Minnesota, USA

**Keywords:** pancreatic neuroendocrine neoplasms, panNENs, pancreatic neuroendocrine tumor, panNET, pNET, pancreatic neuroendocrine carcinoma, panNEC, ABO, blood group, Lewis antigens

## Abstract

Pancreatic neuroendocrine neoplasms (panNENs) are rare endocrine malignancies with poorly defined risk factors, in contrast to the more common pancreatic ductal adenocarcinoma (PDAC), for which non-O ABO blood group is a well-established susceptibility marker. To determine whether ABO and related glycosylation markers associated with PDAC risk extend to panNENs, we conducted a case–control study of 1,059 pathologically confirmed panNEN cases from the Mayo Clinic Biospecimen Resource for Pancreas Research and 33,018 cancer-free controls from the Mayo Clinic Biobank. Using germline genotype data, we inferred ABO blood group, *FUT2*-defined secretor status, and *FUT3*-defined Lewis antigen phenotypes and calculated odds ratios (ORs) and 95% confidence intervals (CIs) using logistic regression adjusted for age, sex, and ancestry principal components. ABO blood group was not associated with panNEN risk: compared with blood group O, the OR for non-O was 0.98 (95% CI: 0.87–1.11), with similarly null findings for blood groups A, B, and AB individually. Secretor status (OR = 0.98, 95% CI: 0.84–1.14) and Lewis-positive phenotypes (OR = 0.91, 95% CI: 0.75–1.13) were also not associated with risk, and no interactions were observed between blood group and secretor status or Lewis antigen phenotypes. Given our high statistical power to detect previously reported PDAC effect sizes for non-O blood group, these null findings are informative, indicating that the ABO-linked glycosylation, inflammatory, and host–microbe interaction pathways implicated in PDAC are unlikely to be major determinants of panNEN susceptibility. This study therefore sharpens the etiologic distinction between pancreatic exocrine and neuroendocrine cancers, redirecting panNEN research beyond PDAC paradigms toward alternative mechanisms of endocrine tumorigenesis.

## Introduction

Pancreatic neuroendocrine neoplasms (panNENs) are rare malignancies of the endocrine pancreas and the second most common pancreatic cancer type after pancreatic ductal adenocarcinoma (PDAC) (1, 2). Although panNENs account for only 1–2% of all pancreatic cancers, compared with about 95% for PDAC, panNENs appear to be biologically and clinically distinct and generally have a more indolent course and better overall survival than PDAC (1, 2, 3, 4, 5). PanNENs comprise pancreatic neuroendocrine tumors (panNETs) and pancreatic neuroendocrine carcinomas (panNECs), which are classified by tumor grade and Ki-67 proliferative index, with panNECs (<10% of panNENs) being poorly differentiated tumors and more aggressive (6, 7). Despite their rarity, the incidence of panNENs has increased in recent decades, likely reflecting improved detection, greater use of cross-sectional imaging leading to incidental diagnoses, and possibly rising prevalence of the underlying risk factors (3, 8, 9).

The lifestyle, environmental, and host genetic determinants of panNENs remain poorly defined and far less studied than PDAC. Emerging evidence suggests that panNEN risk factors may differ from those of PDAC, but findings for alcohol intake, obesity, smoking, and long-standing type 2 diabetes have varied widely across studies, likely reflecting differences in exposure assessment, small sample sizes, and variation in study design (10, 11, 12, 13, 14, 15, 16, 17). These discrepancies underscore the need for complementary approaches to risk assessment in panNENs. Germline genetics may help fill this gap by clarifying inherited susceptibility and potential biological mechanisms underlying panNEN development.

ABO blood group, which can be genetically inferred (18), is a firmly established risk factor for PDAC, with non-O blood groups associated with a higher risk (18, 19, 20, 21, 22). Whether ABO blood group also influences panNEN susceptibility remains unclear. A small Han Chinese case–control study found no association (23), whereas two small studies in patients with von Hippel–Lindau (VHL) (24) or multiple endocrine neoplasia type 1 (MEN1) (25) syndrome suggested a possible association. However, their limited sample sizes and highly selected patient populations restrict generalizability. These preliminary and conflicting findings provide the impetus for a larger, more comprehensive evaluation of the association between ABO blood group and panNEN risk.

Although panNENs exhibit neuroendocrine differentiation and are pathologically distinct from PDAC, they are classified by the World Health Organization (WHO) as epithelial neuroendocrine neoplasms (7). Moreover, ABO-related glycosylation may influence cancer susceptibility beyond the tumor cell of origin through local and systemic mechanisms, including immune surveillance, inflammation, intercellular adhesion, and membrane signaling (26). Determining whether the ABO-associated risk observed for PDAC extends to panNENs, and whether related glycosylation markers, including secretor status and Lewis antigens, influence panNEN susceptibility, could advance our understanding of the etiology of this distinct pancreatic malignancy.

The ABO histo-blood group antigens are expressed on mucosal epithelia of the gastrointestinal, genitourinary, and respiratory tracts, sites that can function as host receptor interfaces for microbial adhesion and infection, and the antigens are also expressed on platelets (22, 27). Biosynthesis and tissue expression of these antigens are coordinated by activities of the *ABO, FUT2* (secretor), and *FUT3* (Lewis) genes (22, 27). The ABO locus encodes a glycosyltransferase that modifies the H antigen precursor, synthesized through *FUT1* activity, by adding allele-specific sugar residues to generate the A or B antigens. By contrast, the O allele encodes a nonfunctional enzyme that leaves the H antigen unmodified. These alleles are tagged by single nucleotide variants (SNVs) at the *ABO* locus (19, 20, 21).

Secretor status is determined by a *FUT2* SNV encoding a fucosyltransferase required for the secretion of ABO antigens into gastrointestinal fluids and their expression on mucosal epithelia (28), providing a plausible basis for the effect modification of ABO-associated disease risk by secretor status. *FUT3* encodes a fucosyltransferase essential for Lewis antigen synthesis, and these antigens are initially produced on mucosal surfaces and can later be detected on red blood cells (29). Variation in *FUT3* determines the principal Lewis phenotypes (Lewis-positive normal, Lewis-positive semi-normal, and Lewis-negative) (22).

Although the association between ABO blood group and panNEN risk has been explored in a few small studies, the potential roles of *FUT2* (secretor status) and *FUT3* (Lewis antigens) have not, to our knowledge, been evaluated, nor have potential interactions among *ABO, FUT2,* and *FUT3* genotypes been assessed regarding panNEN susceptibility. We hypothesized that genetically inferred ABO blood group and related glycosylation phenotypes, including *FUT2*-defined secretor status and *FUT3*-defined Lewis antigen phenotypes, are associated with panNEN susceptibility. We therefore evaluated these associations with panNEN risk and examined whether any association with ABO blood group varies by secretor status or Lewis antigen activity.

## Materials and methods

### Study population and data collection

Germline genetic data and risk factor information were obtained from two Mayo Clinic resources that draw from the same source population: the Mayo Clinic Biospecimen Resource for Pancreas Research (cases) and the Mayo Clinic Biobank (controls). The design and recruitment processes for these data sources have been described in detail (18, 30, 31, 32). Briefly, the Mayo Clinic Biospecimen Resource for Pancreas Research uses an ultra-rapid case finding process and has enrolled participants since October 2000. Potentially eligible patients are contacted for consent by telephone before their clinic visit for the evaluation of suspected pancreatic cancer or during their initial clinic appointment. Between October 2000 and August 2023, 1,886 patients diagnosed with panNENs were approached and 1,298 consented to the registry (69% response rate). Among enrolled cases, 55% were recruited within 30 days of diagnosis, with an average of 16 days between first contact and enrollment (10). Cases were eligible if they were ≥18 years or older, had a pathologically confirmed diagnosis of incident panNENs, and had provided peripheral blood samples that were used for genotyping by the Regeneron Genetics Center (RGC). Because tumor grade and/or Ki-67 proliferation index were unavailable for a substantial proportion of participants (∼40%), we were unable to reliably distinguish panNETs (expected to be >90%) from panNECs for many cases; hence, panNENs were analyzed as a single phenotype. Controls were drawn from the Mayo Clinic Biobank, which has enrolled participants from primary care and specialty clinics (e.g. orthopedics, obstetrics/gynecology, and executive health) starting April 2009 (32). Eligibility criteria included age ≥18 years, US residence, and ability to provide informed consent. The biobank has enrolled over 56,000 participants with a response rate of 21% (32). For the present study, we included biobank controls without a history of cancer (except nonmelanoma skin cancer) who had provided peripheral blood samples that were used for genotyping by RGC.

Cases and controls completed similar risk factor questionnaires soliciting information on demographics (e.g. age and sex), anthropometrics (usual adult weight and height), cigarette smoking, alcohol intake, and first-degree family history of pancreatic cancer. Clinical data, including type 2 diabetes mellitus (T2DM), were abstracted from participant medical records. Body mass index (BMI) was calculated as weight divided by height in kg/m^2^ using self-reported usual adult weight and height. Smoking status was categorized as never (≤100 cigarettes in a lifetime), former, or current. Alcohol intake and T2DM were treated as binary variables (ever/never or yes/no).

### Genotyping

Germline DNA extracted from peripheral blood samples of cases and controls was sequenced in two batches, with each batch containing a mix of cases and controls, at the RGC (Tarrytown, NY, USA) using a custom target capture panel as part of the Mayo Clinic Project Generation initiative (33, 34, 35). The assay was designed to interrogate exonic regions while also providing a broad coverage of common variants via the genotyping-by-sequencing framework (33). Standard variant- and sample-level quality control (QC) procedures were applied simultaneously to samples from both genotyping batches. Assessment of batch effects showed no evidence of systematic differences between batches. Common variants (SNVs) were removed if they did not satisfy prespecified thresholds for call rate (<95%), minor allele frequency (MAF < 0.5%), or Hardy–Weinberg equilibrium (*P* < 1 × 10^−6^) (33, 34, 35). Individuals were excluded for excessive missingness (>5% missing genotypes), discordance between genetically determined and self-reported sex, or abnormal heterozygosity (<70% across multiple chromosomes). Relatedness filtering was performed using iterative kinship assessment with PLINK and PRIMUS, excluding participants with evidence of excess kinship (PI_HAT > 0.1875 with >100 relatives or PI_HAT > 0.08 with >25,000 relatives). When a second-degree or closer relationship was detected between a case and a control, the case subject was retained and the control was removed, and when relatedness occurred within case–case or control–control pairs, we retained the individual with more complete covariate data (35). Genotype imputation was performed using the TOPMed Imputation Server (36, 37). Post-imputation, only well-imputed variants (dosage *R*^2^ > 0.8) with MAF ≥5% were retained. After QC, the final analytic data comprised 1,059 panNEN cases and 33,018 controls (details in Supplementary Table 1 (see section on Supplementary materials given at the end of the article)). Genetic ancestry was inferred using principal components calculated from 4,874 high-quality ancestry-informative SNVs that overlap with HapMap3. Individuals were then classified into continental ancestry groups (African, Admixed American, East Asian, European, and South Asian) by projecting them onto HapMap3 reference principal components and assigning ancestry using the maximum likelihood method.

### Genotype-derived ABO, secretor, and Lewis antigen phenotypes

We list in Supplementary Table 2 the variants and inference rules used to derive genetically inferred ABO blood groups and genotypes corresponding to secretor status and Lewis antigen activity. ABO alleles (A, B, and O) were inferred using haplotypes defined by rs505922 (O vs A allele), rs8176746/rs8176693 (B allele), and rs574347 (A allele) (19, 20, 21, 22). Due to missing genotype data for rs8176746 in a subset of participants, rs8176693 was used as a proxy to tag the B allele because the two SNPs are in near-perfect linkage disequilibrium (*r*^2^ ≥ 0.99) across major 1000 Genomes Phase III populations (CEU, ASW, CHB, CHD, and YRI) (38, 39, 40). Secretor status was inferred using *FUT2* rs601338 (G>A), with individuals homozygous for the A allele classified as non-secretors (19, 22). Lewis antigen activity was inferred from three *FUT3* variants (rs28362459, rs812936, and rs3894326) to classify participants as Lewis-positive normal, Lewis-positive semi-normal, and Lewis-negative (22, 41). Using this approach, we derived ABO alleles (A, B, AB, and O), *FUT2*-based secretor status (secretor vs non-secretor, or unknown for missing genotypes), and *FUT3*-based Lewis antigen groups (Lewis-positives and Lewis-negative) for analyses.

### Statistical analysis

Participant characteristics were compared between cases and controls using ANOVA for continuous variables and chi-square tests for categorical variables. Unconditional logistic regression was used to assess associations of ABO blood group, secretor status, and Lewis antigen variation with panNEN risk, calculating odds ratios (ORs) and 95% confidence intervals (CIs) in unadjusted and minimally adjusted models. The adjusted models included age (continuous), sex, and the top 10 principal components of genetic ancestry. To assess whether the association between ABO blood group and panNEN risk differed by secretor status or Lewis antigen activity, we conducted stratified analyses of the association between ABO blood group (non-O vs O) and panNEN risk separately among secretors and non-secretors, and separately among participants with Lewis antigen positives (Lewis-positive normal + semi-normal) and those with Lewis antigen negative. We also evaluated interactions between blood group (non-O vs O) and secretor status (secretors vs non-secretors) or Lewis antigen phenotype (Lewis-positives vs Lewis-negative) using likelihood ratio tests. Statistical power was estimated *post hoc* based on the final analytic sample of 1,059 panNEN cases and 33,018 controls, assuming effect sizes previously reported for PDAC (ORs for non-O vs O blood group ranging from 1.28 to 1.50) (20, 21, 22, 42) and using logistic regression modeling with a two-sided *α* = 0.05 (Supplementary Table 3). In sensitivity analyses, we evaluated the associations of ABO blood group, secretor status, and Lewis antigen variation with panNEN risk using fully adjusted models that included age (continuous), sex, the top 10 ancestry principal components, T2DM (yes/no), BMI (continuous), smoking status (never, former, current), and alcohol intake (non-user, <1 drink/day, 1–2 drinks/day, ≥3 drinks/day). We also repeated these analyses after restricting cases to patients with clinically annotated nonfunctional panNENs (*n* = 897; i.e. not causing hormone-related symptoms), using the same minimally and fully adjusted models. We could not perform a corresponding analysis among patients with functional panNENs because the small number of cases (*n* = 162) precluded stable estimates. All analyses were conducted using R, version 4.4.1 (R Foundation for Statistical Computing, Austria), and SAS, version 9.4 (SAS Institute Inc., USA). Statistical power was calculated using nQuery Advisor, version 7.0.

## Results

Distributions of demographic and risk factor characteristics for the 1,059 panNEN cases and 33,018 cancer-free controls are shown in Table 1. Briefly, compared with controls, cases were on average two years older and more likely to be male, have T2DM, and report alcohol use. The two groups were otherwise similar with respect to BMI, genetic ancestry, and smoking history.

**Table 1:** Descriptive characteristics of study participants (*n* = 34,111).

	panNEN cases (*n* = 1059)	Controls (*n* = 33,018)	*P* value^‡^
Age in years, mean (SD)*	59.7 (12.7)	57.7 (15.2)	<0.001
Sex			<0.001
Male	586 (55.3%)	13,322 (40.3%)	
Female	473 (44.7%)	19,696 (59.7%)	
BMI, kg/m^2^			0.527
Mean (SD)	28.7 (5.749)	28.8 (6.098)	
Unknown	1	269	
Genetic ancestry^†^			0.112
Admixed American	18 (1.7%)	319 (1.0%)	
African	10 (0.9%)	492 (1.5%)	
East Asian	6 (0.6%)	266 (0.8%)	
European	1017 (96.0%)	31,627 (95.8%)	
South Asian	4 (0.4%)	178 (0.5%)	
Unknown	4 (0.4%)	136 (0.4%)	
Self-reported DM			<0.001
No	688 (78.6%)	27,659 (83.8%)	
Yes	187 (21.4%)	5,359 (16.2%)	
Unknown	184	0	
Alcohol use			<0.001
Ever	535 (73.0%)	25,981 (79.8%)	
Never	198 (27.0%)	6,596 (20.2%)	
Unknown	326	441	
Cigarette smoking			0.389
Never	601 (57.9%)	19,635 (60.0%)	
Former	381 (36.7%)	11,401 (34.9%)	
Current	56 (5.4%)	1677 (5.1%)	
Unknown	21	306	

* Age at diagnosis for cases and at recruitment for controls.

^†^ Ancestry was determined using principal components from 4,874 ancestry-informative single nucleotide variants overlapping HapMap3.

^‡^ *P*-values were calculated using analysis of variance (ANOVA) for continuous variables and Pearson’s chi-squared tests for categorical variables.

BMI, body mass index; DM, diabetes mellitus; panNENs, pancreatic neuroendocrine neoplasms.

**Figure 1:**
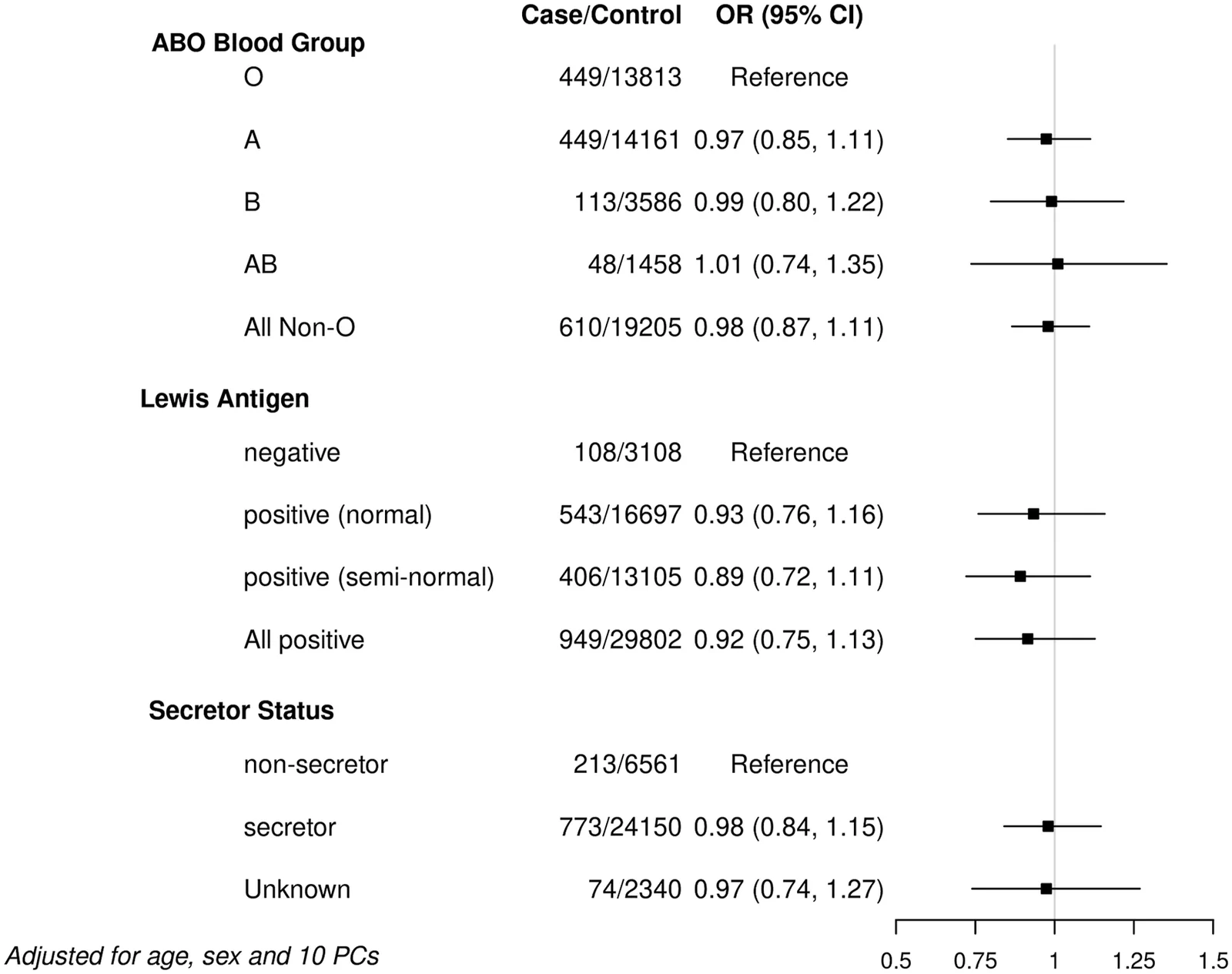
Association of ABO blood group, secretor status, and Lewis antigen phenotypes with risk of pancreatic neuroendocrine neoplasms, adjusted for age (continuous), sex, and the top 10 principal components of genetic ancestry. No significant associations were observed between the ABO glycosylation markers and risk of pancreatic neuroendocrine neoplasms.

In both the unadjusted models (Supplementary Fig. 1) and minimally adjusted models that included age, sex, and ancestry principal components (Fig. 1), we found no evidence that ABO blood group was associated with panNEN risk. Compared with blood group O, blood groups A, B, and AB neither individually nor collectively as non-O blood group were associated with risk. In the adjusted model, the OR for non-O vs O was 0.98 (95% CI: 0.87–1.11) (Fig. 1). Secretor status was likewise not associated with panNEN risk (secretors *vs* non-secretors: OR = 0.98; 95% CI: 0.84–1.14). Similarly, Lewis antigen phenotype was not associated with risk: compared with Lewis-negative individuals, the ORs were 0.93 (95% CI: 0.76–1.16) for Lewis-positive normal, 0.89 (95% CI: 0.72–1.11) for Lewis-positive semi-normal, and 0.91 (95% CI: 0.75–1.13) for the combined Lewis-positive normal and semi-normal phenotype (Fig. 1). *Post hoc* power calculations indicated that, given the available sample size, the study had ≥97% power to detect an association for non-O versus O blood group at previously reported PDAC effect sizes of OR ≥1.28 (Supplementary Table 3).

**Figure 2:**
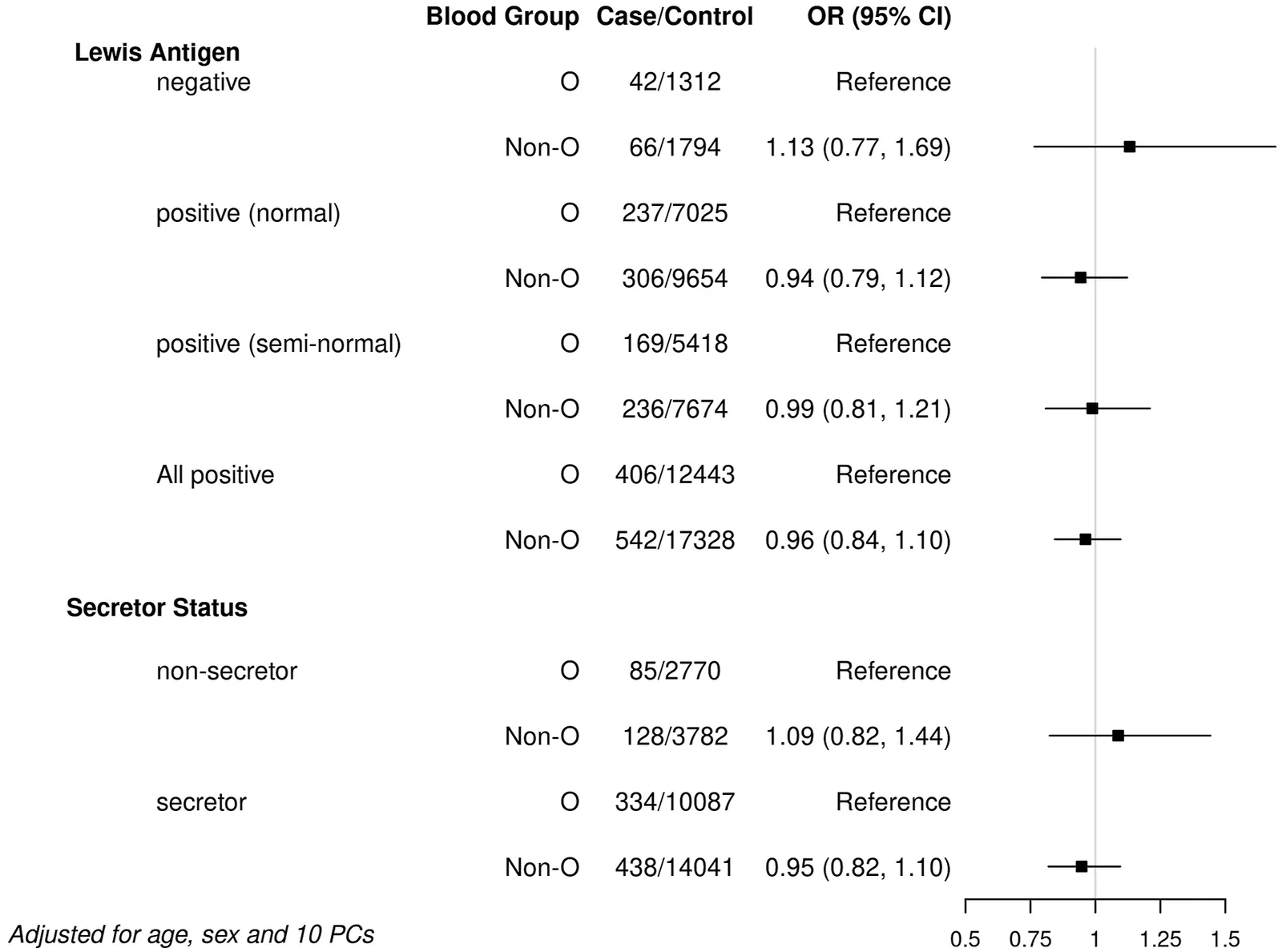
Association of non-O ABO blood group with risk of pancreatic neuroendocrine neoplasms, adjusted for age (continuous), sex, and the top 10 principal components of genetic ancestry, and stratified by secretor status and Lewis antigen phenotype. No evidence of effect modification by secretor status or Lewis antigen phenotype was observed.

Stratified analyses showed no association between non-O blood group and panNEN risk by secretor status (Fig. 2; Supplementary Fig. 2). In the adjusted models, compared with blood group O, the ORs for non-O blood group were 0.95 (95% CI: 0.82–1.10) among secretors and 1.09 (95% CI: 0.82–1.44) among non-secretors (*P* for interaction = 0.44) (Fig. 2). Similarly, in analyses stratified by Lewis phenotype, non-O blood group was not associated with panNEN risk among Lewis-negative individuals (OR = 1.13, 95% CI: 0.77–1.69), those with the Lewis-positive normal phenotype (OR = 0.94, 95% CI: 0.79–1.12), those with the Lewis-positive semi-normal phenotype (OR = 0.99, 95% CI: 0.81–1.21), or those with the combined Lewis-positive normal and semi-normal phenotype (OR = 0.96, 95% CI: 0.84–1.10) (Fig. 2); all interaction *P* values ≥ 0.48.

In sensitivity analyses, we found no evidence of associations between ABO blood group, secretor status, or Lewis antigen variation and panNEN risk in fully adjusted models that included age, sex, the top 10 ancestry principal components, T2DM, BMI, smoking status, and alcohol intake (Supplementary Fig. 3). Similarly, analyses restricted to patients with clinically annotated nonfunctional panNENs showed no associations with ABO blood group, secretor status, or Lewis antigen variation in either the minimally adjusted model (Supplementary Fig. 4) or the fully adjusted model (Supplementary Fig. 5).

## Discussion

In this study, we found no evidence that genetically inferred ABO blood group or related glycosylation markers are associated with panNEN risk. Compared with blood group O, neither non-O blood groups overall nor A, B, and AB blood groups individually were associated with risk, and associations were similarly null for secretor status and Lewis antigen phenotypes. We also found no evidence of effect modification by secretor status or Lewis phenotype. Together, these findings suggest that the well-established ABO-related susceptibility observed for PDAC does not extend to panNENs and underscore important etiologic differences between pancreatic exocrine and neuroendocrine malignancies.

Prior studies of association between ABO blood group and panNENs have been sparse and heterogeneous, consisting mainly of small hereditary syndrome cohorts (24, 25, 43), a small case–control study that found no association with panNEN risk (23), and a post-operative case series suggesting a possible association with recurrence after panNEN resection (44). Our study, the largest and most comprehensive to date, extends prior work by evaluating not only ABO blood group but also related secretor status and Lewis antigen phenotypes. Our findings align with those of Ben *et al.* who observed no association between ABO blood group and risk in a hospital-based case–control study of sporadic panNETs (23). By contrast, previous *VHL* (24) and *MEN1* (25) studies examined ABO blood group distributions in highly selective hereditary syndrome cohorts and did not perform formal risk association analyses and were limited by small sample sizes. Notably, the *MEN1* finding was not replicated in a subsequent Dutch *MEN1* cohort (43). These data and our results collectively suggest that ABO-related glycosylation is unlikely to be a major determinant of panNEN susceptibility.

Although the biological basis for a link between ABO blood group and cancer remains uncertain, several mechanisms make such an association plausible. ABO antigens and the glycosyltransferases that generate them may influence epithelial and platelet biology, including cell adhesion, membrane signaling, immune recognition, and interactions with microbes (22, 27). Variation in ABO-related glycosylation could affect tumor development indirectly through inflammatory pathways, as genetic variants at the ABO locus have been associated with circulating levels of inflammatory markers, such as TNF-α and intercellular adhesion molecule-1 (45, 46). Because *FUT2*-dependent secretor status and *FUT3*-dependent Lewis antigens also shaped tissue expression of these glycans, related glycosylation phenotypes were thought to be likewise relevant to panNEN risk (22, 47, 48). These factors, together with the established association between ABO and PDAC risk (19, 20, 21, 22, 49), provided the rationale for examining ABO blood group, secretor status, and Lewis phenotypes in relation to panNEN risk.

Our study has several strengths, including its large sample size for a rare malignancy that is well powered for effect sizes previously reported for PDAC, pathologically confirmed cases recruited through an ultra-rapid ascertainment process, and the use of cases and controls drawn from the same source population with germline data generated on a common platform. The genotype data also enabled evaluation of ABO blood group alongside *FUT2*-inferred secretor status and *FUT3*-inferred Lewis phenotypes. Several limitations merit consideration. Because cases and controls were recruited from a tertiary referral hospital rather than a population-based sampling frame, selection bias and limited generalizability are possible. Referral patterns to our tertiary hospital may have preferentially captured patients with more complex, advanced, or atypical disease and may have differed between cases and controls. Consequently, the study population may not fully represent patients with panNENs treated in community- or population-based settings. Because approximately 96% of participants were of European ancestry, the generalizability of our findings to other ancestral populations may be limited, particularly given the population differences in the frequencies of *ABO, FUT2*, and *FUT3* alleles and their corresponding phenotypes. Replication in more ancestrally diverse cohorts is therefore warranted. In addition, ABO, secretor status, and Lewis antigen phenotypes were genetically inferred rather than directly measured by serology or tissue. Furthermore, incomplete clinicopathologic annotation, including tumor grade and Ki-67 index, for a substantial proportion of cases precluded reliable analyses by tumor grade or separate evaluation of panNETs and panNECs. Although we performed additional analyses restricted to clinically annotated nonfunctional panNENs, the number of functional tumors was insufficient for a meaningful subgroup analysis. Therefore, associations confined to specific histologic, grade, or functional subgroups cannot be excluded. Also, the null findings observed in this study should be interpreted within the study’s scope. Although our results argue against moderate overall associations comparable to those reported for PDAC, smaller effect sizes cannot be excluded. For the primary comparison of non-O versus O blood group, the study had 97% power to detect an OR of 1.28 and >99% power to detect an OR of 1.50, encompassing the range of associations previously reported for PDAC. The approximate minimum detectable OR at 80% power is 1.20. Thus, our findings argue against a moderate overall effect comparable to that reported for PDAC but do not exclude smaller associations. Such effects could remain etiologically relevant at the population level, although their utility for individual clinical risk prediction would likely be limited. Data on diabetes and alcohol intake were missing for some participants, and the underlying mechanism of missingness could not be determined. However, complete case sensitivity analyses incorporating these and other covariates produced results consistent with the primary analyses. Finally, because our study evaluated panNEN susceptibility rather than clinical outcomes, potential associations with disease progression or prognosis were not assessed.

In this study, we found no evidence that genetically inferred ABO blood group, secretor status, or Lewis antigen phenotype is associated with the risk of panNEN, nor that these glycosylation-related traits interact to influence susceptibility. These findings suggest that ABO, despite its established role in PDAC, is unlikely to be a major determinant of panNEN risk and further support distinct etiologic pathways underlying pancreatic exocrine and neuroendocrine neoplasms. Future studies in more diverse populations and with detailed tumor characterization may help clarify alternative biological mechanisms driving panNEN development.

## Supplementary materials













## Declaration of interest

The authors declare that there is no conflict of interest that could be perceived as prejudicing the impartiality of the work reported.

## Funding

This work was supported by NIH (Grant Nos. K01CA237875 and P30CA15083) and the Genetic Epidemiology Risk Assessment for Pancreatic Cancer initiative honoring Dr Gloria Petersen.

## Ethical statement

This study was approved by the Mayo Clinic Institutional Review Board (Protocol #22–001158) and conducted in accordance with the Declaration of Helsinki and the Declaration of Istanbul.

## Data availability

Summary-level data supporting the findings of this study for the validation analyses are available from the corresponding author upon reasonable request, subject to institutional and contractual approvals. Individual-level genetic data from the Project Generation cohort used for validation analyses are subject to contractual restrictions and are not publicly available. These data were generated by the Regeneron Genetics Center as part of a collaboration with Mayo Clinic and may be accessed only by approved investigators within secure Mayo Clinic computing environments, in accordance with institutional and data use agreements.

## AI disclosure

No generative artificial intelligence (AI) tools were used in the preparation of the written or visual content of this manuscript.

## Collaborator/consortium banner authors

James R Cerhan¹, Fergus J Couch¹, Nicholas B Larson¹, Eric W Klee¹, Zachary S Fredericksen¹, Mine Cicek¹, Janet E Olson¹, Lisa K Colborn¹, Andrew J Danielsen¹, Jonathan J Harrington¹, Aris Baras², Gonçalo Abecasis², Adolfo Ferrando², Giovanni Coppola², Andrew Deubler², Luca A Lotta², John D Overton², Jeffrey G Reid², Alan Shuldiner², Katherine Siminovitch², Jason Portnoy², Marcus B Jones², Lyndon Mitnaul², Alison Fenney², Jonathan Marchini², Manuel Allen Revez Ferreira², Maya Ghoussaini², Mona Nafde², William Salerno², Cristen Willer², Lourdes Crane², Christina Beechert², Erin Fuller², Laura M Cremona², Eugene Kalyuskin², Hang Du², Caitlin Forsythe², Zhenhua Gu², Kristy Guevara², Michael Lattari², Alexander Lopez², Kia Manoochehri², Prathyusha Challa², Manasi Pradhan², Raymond Reynoso², Ricardo Schiavo², Maria Sotiropoulos Padilla², Chenggu Wang², Sarah E Wolf², Jeffrey G Reid², Mona Nafde², Manan Goyal², George Mitra², Sanjay Sreeram,² Rouel Lanche,² Vrushali Mahajan,² Sai Lakshmi Vasireddy,² Gisu Eom,² Krishna Pawan Punuru,² Sujit Gokhale,² Benjamin Sultan,² Pooja Mule,² Mudasar Sarwar,² Muhammad Aqeel,² Xiaodong Bai,² Lance Zhang,² Sean O’Keeffe,² Razvan Panea,² Evan Edelstein,² Ayesha Rasool,² Evan K Maxwell,² Boris Boutkov,² Alexander Gorovits,² Ju Guan,² Lukas Habegger,² Alicia Hawes,² Olga Krasheninina,² Samantha Zarate,² Adam J Mansfield,² Joshua Backman,² Kathy Burch,² Adrian Campos,² Liron Ganel,² Sheila Gaynor,² Benjamin Geraghty,² Arkopravo Ghosh,² Salvador Romero Martinez,² Christopher Gillies,² Lauren Gurski,² Eric Jorgenson,² Tyler Joseph,² Michael Kessler,² Jack Kosmicki,² Adam Locke,² Priyanka Nakka,² Karl Landheer,² Olivier Delaneau,² Anthony Marcketta,² Joelle Mbatchou,² Arden Moscati,² Anita Pandit,² Jonathan Ross,² Carlo Sidore,² Eli Stahl,² Timothy Thornton,² Sailaja Vedantam,² Rujin Wang,² Kuan-Han Wu,² Bin Ye,² Blair Zhang,² Andrey Ziyatdinov,² Yuxin Zou,² Jingning Zhang,² Kyoko Watanabe,² Mira Tang,² Frank Wendt,² Suganthi Balasubramanian,² Suying Bao,² Kathie Sun,² Chuanyi Zhang,² Sean Yu,² Aaron Zhang,² David Corrigan,² Dhruv Shidhaye,² Chen Wang,² Keyrun Adhikari,² Alexander Lachmann,² Brian Hobbs,² Jon Silver,² William Palmer,² Rita Guerreiro,² Amit Joshi,² Antoine Baldassari,² Sarah Graham,² Ernst Mayerhofer,² Erola Pairo Castineira,² Mary Haas,² Niek Verweij,² George Hindy,² Jonas Bovijn,² Tanima De,² Luanluan Sun,² Olukayode Sosina,² Arthur Gilly,² Peter Dornbos,² Juan Rodriguez-Flores,² Moeen Riaz,² Manav Kapoor,² Gannie Tzoneva,² Momodou W Jallow,² Anna Alkelai,² Ariane Ayer,² Veera Rajagopal,² Sahar Gelfman,² Vijay Kumar,² Jacqueline Otto,² Jose Bras,² Silvia Alvarez,² Jessie Brown,² Hossein Khiabanian,² Joana Revez,² Kimberly Skead,² Valentina Zavala,² Jae Soon Sul,² Lei Chen,² Sam Choi,² Amy Damask,² Nan Lin,² Charles Paulding,² Sameer Malhotra,² Joseph Herman,² Michelle G LeBlanc,² Nadia Rana,² Jennifer Rico-Varela,² Jaimee Hernandez,² Larizbeth Romero,² Ashley Paynter,² Randi Schwartz,² Jody Hankins,² Anna Han,² Samuel Hart,² Ryan Smith,² Ann Perez-Beals,² Gina Solari,² Johannie Rivera-Picart,² Michelle Pagan,² Sunilbe Siceron². Affiliations: ¹Department of Quantitative Health Sciences, Mayo Clinic, Rochester, MN, USA; ²Regeneron Genetics Center, Tarrytown, NY, USA.
