# Supplementary material for: Genetically determined ABO blood group, secretor status, Lewis antigens, and panNEN risk
Source: Endocr Relat Cancer. 2026 Sep 18;33(9):e260286. doi: 10.1530/ERC-26-0286 (PMC13615866; doi:10.1530/ERC-26-0286)

	Case/Control	OR (95% CI)
**ABO Blood Group**		
O	449/13813	Reference
A	449/14161	0.98 (0.85, 1.11)
B	113/3586	0.97 (0.78, 1.19)
AB	48/1458	1.01 (0.74, 1.36)
All Non-O	610/19205	0.98 (0.86, 1.11)
**Lewis Antigen**		
negative	108/3108	Reference
positive (normal)	543/16697	0.94 (0.76, 1.16)
positive (semi-normal)	406/13105	0.89 (0.72, 1.11)
All positive	949/29802	0.92 (0.75, 1.13)
**Secretor Status**		
non-secretor	213/6561	Reference
secretor	773/24150	0.99 (0.85, 1.15)
Unknown	74/2340	0.97 (0.74, 1.27)

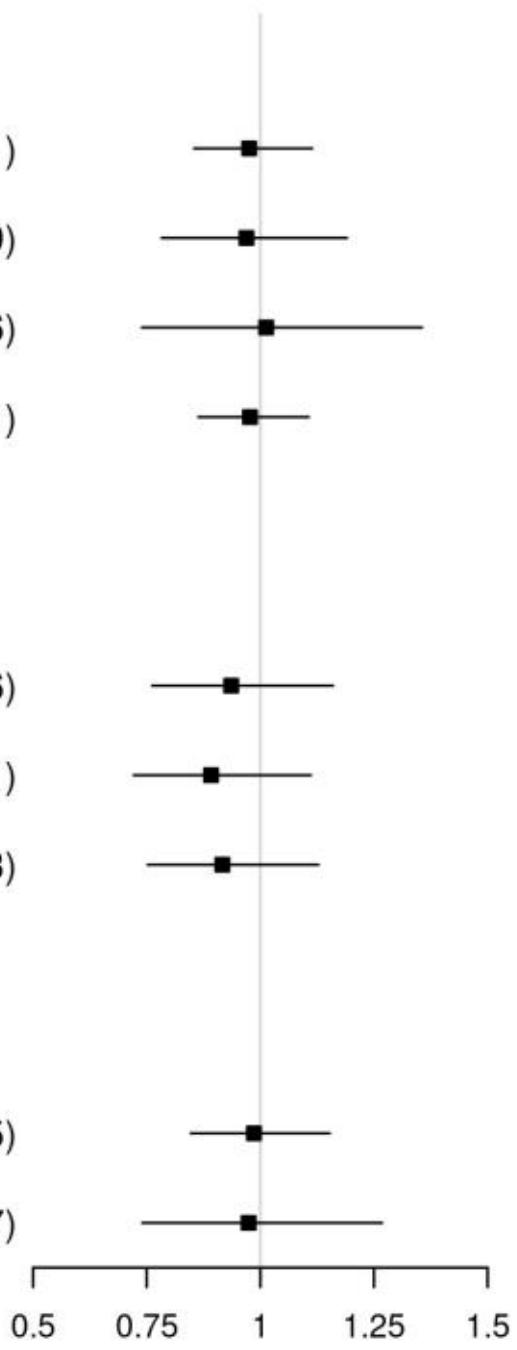

Supplement: Supplementary file 1 [file ERC-26-0286_supplementary_figure_1.pdf]
