# Supplementary material for: Genetically determined ABO blood group, secretor status, Lewis antigens, and panNEN risk
Source: Endocr Relat Cancer. 2026 Sep 18;33(9):e260286. doi: 10.1530/ERC-26-0286 (PMC13615866; doi:10.1530/ERC-26-0286)

	Blood Group	Case/Control	OR (95% CI)
**Lewis Antigen**			
negative	O	42/1312	Reference
	Non-O	66/1794	1.15 (0.78, 1.71)
positive (normal)	O	237/7025	Reference
	Non-O	306/9654	0.94 (0.79, 1.12)
positive (semi-normal)	O	169/5418	Reference
	Non-O	236/7674	0.99 (0.81, 1.21)
All positive	O	406/12443	Reference
	Non-O	542/17328	0.96 (0.84, 1.09)
**Secretor Status**			
non-secretor	O	85/2770	Reference
	Non-O	128/3782	1.10 (0.84, 1.46)
secretor	O	334/10087	Reference
	Non-O	438/14041	0.94 (0.82, 1.09)

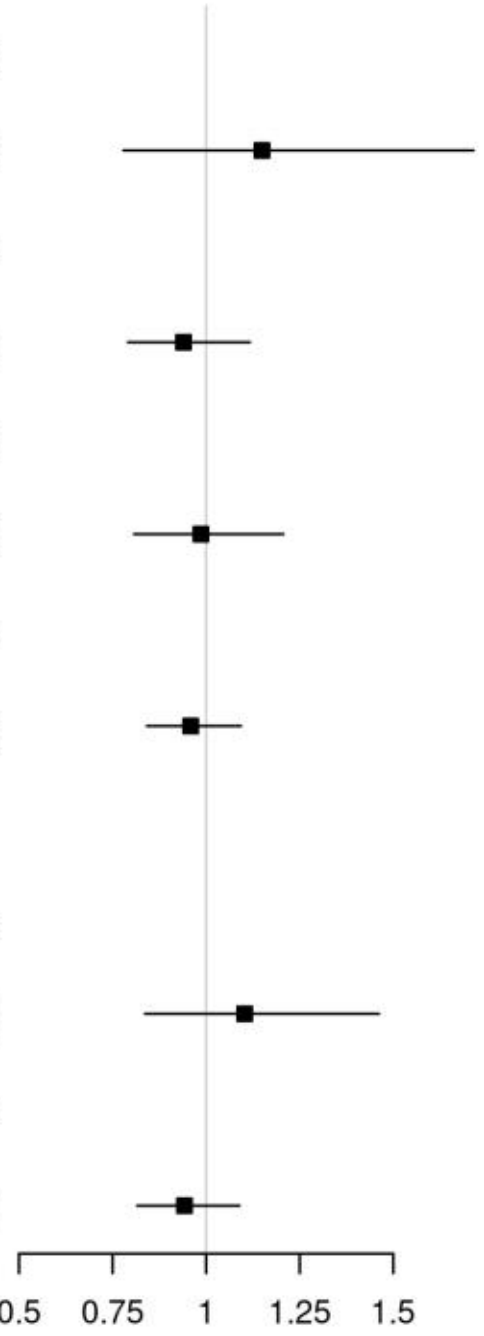

Supplement: Supplementary file 2 [file ERC-26-0286_supplementary_figure_2.pdf]
