# Supplementary material for: Genetically determined ABO blood group, secretor status, Lewis antigens, and panNEN risk
Source: Endocr Relat Cancer. 2026 Sep 18;33(9):e260286. doi: 10.1530/ERC-26-0286 (PMC13615866; doi:10.1530/ERC-26-0286)
Supplement: Supplementary file 3 [file ERC-26-0286_supplementary_figure_3.pdf]

	Case/Control	OR (95% CI)
**ABO Blood Group**		
O	281/13170	Reference
A	258/13521	0.89 (0.75, 1.06)
B	70/3435	1.00 (0.76, 1.30)
AB	32/1372	1.14 (0.77, 1.63)
All Non-O	360/18328	0.93 (0.79, 1.09)
**Lewis Antigen**		
negative	71/2964	Reference
positive (normal)	327/15902	0.82 (0.64, 1.08)
positive (semi-normal)	241/12499	0.79 (0.61, 1.04)
All positive	568/28401	0.81 (0.63, 1.05)
**Secretor Status**		
non-secretor	137/6232	Reference
secretor	460/23051	0.92 (0.76, 1.13)
Unknown	44/2215	0.94 (0.66, 1.32)

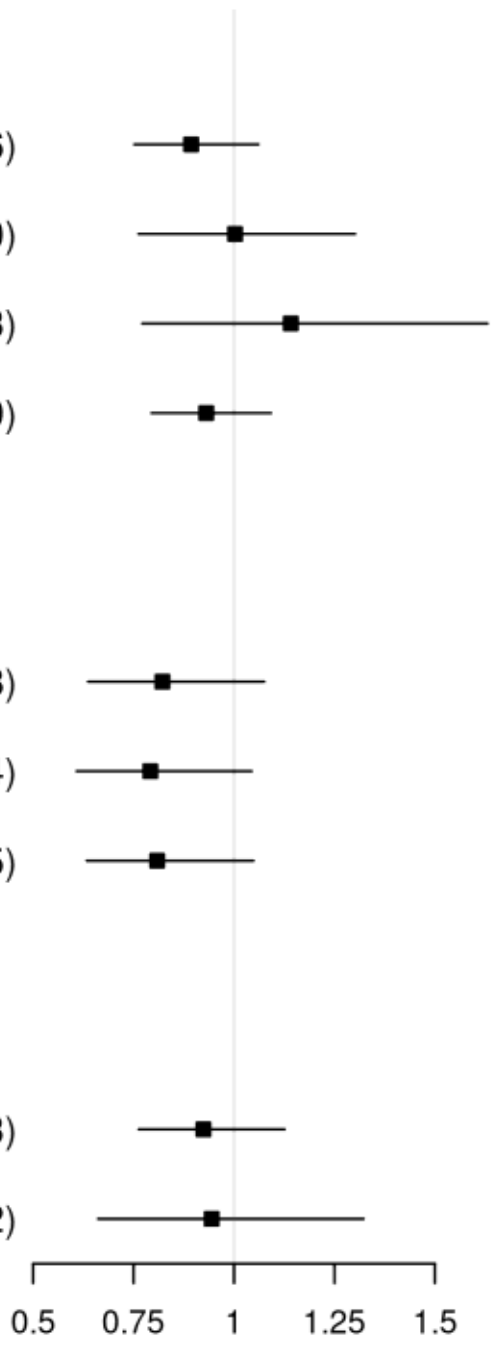

Adjusted for age, sex, diabetes, bmi, smoking status, alcohol use and 10 PCs
