# Supplementary material for: Genetically determined ABO blood group, secretor status, Lewis antigens, and panNEN risk
Source: Endocr Relat Cancer. 2026 Sep 18;33(9):e260286. doi: 10.1530/ERC-26-0286 (PMC13615866; doi:10.1530/ERC-26-0286)
Supplement: Supplementary file 4 [file ERC-26-0286_supplementary_figure_4.pdf]

	Case/Control	OR (95% CI)
**ABO Blood Group**		
O	382/13813	Reference
A	381/14161	0.97 (0.84, 1.12)
B	94/3586	0.97 (0.77, 1.22)
AB	40/1458	0.99 (0.70, 1.36)
All Non-O	515/19205	0.97 (0.85, 1.11)
**Lewis Antigen**		
negative	89/3106	Reference
positive (normal)	449/16679	0.94 (0.75, 1.19)
positive (semi-normal)	357/13092	0.95 (0.76, 1.21)
All positive	806/29771	0.94 (0.76, 1.19)
**Secretor Status**		
non-secretor	168/6552	Reference
secretor	667/24128	1.07 (0.91, 1.28)
Unknown	62/2338	1.04 (0.77, 1.39)

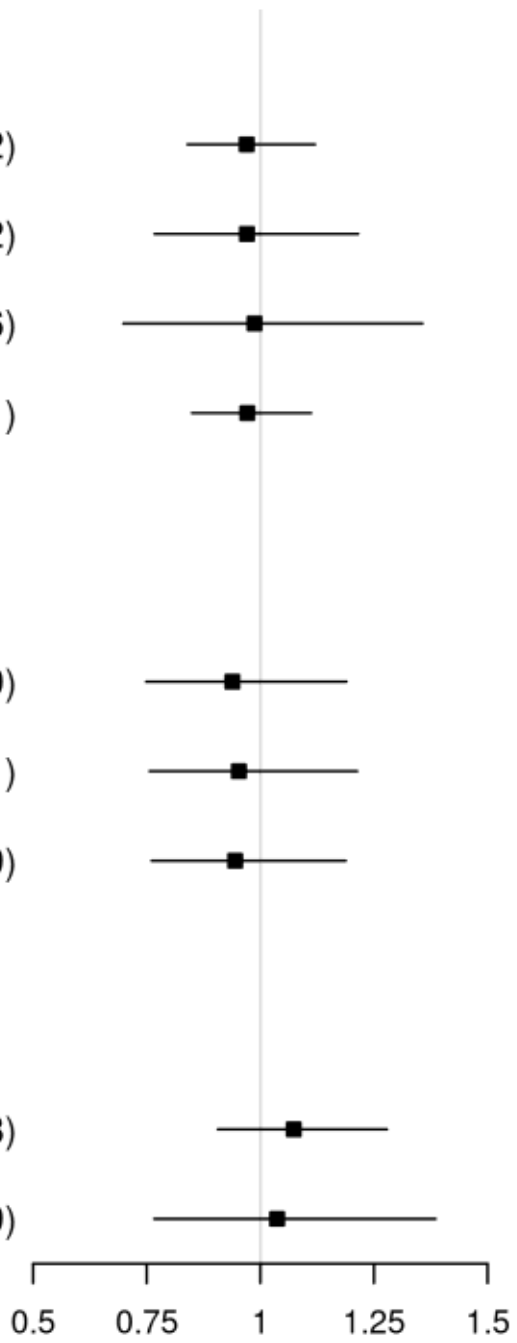

Adjusted for age, sex and 10 PCs
