# Supplementary material for: Genetically determined ABO blood group, secretor status, Lewis antigens, and panNEN risk
Source: Endocr Relat Cancer. 2026 Sep 18;33(9):e260286. doi: 10.1530/ERC-26-0286 (PMC13615866; doi:10.1530/ERC-26-0286)
Supplement: Supplementary file 5 [file ERC-26-0286_supplementary_figure_5.pdf]

	Case/Control	OR (95% CI)
**ABO Blood Group**		
O	230/13170	Reference
A	209/13521	0.88 (0.73, 1.07)
B	57/3435	1.01 (0.74, 1.34)
AB	26/1372	1.13 (0.73, 1.67)
All Non-O	292/18328	0.92 (0.77, 1.10)
**Lewis Antigen**		
negative	60/2964	Reference
positive (normal)	256/15902	0.76 (0.58, 1.02)
positive (semi-normal)	205/12499	0.80 (0.60, 1.07)
All positive	461/28401	0.78 (0.59, 1.03)
**Secretor Status**		
non-secretor	105/6232	Reference
secretor	379/23051	1.00 (0.80, 1.25)
Unknown	38/2215	1.06 (0.72, 1.53)

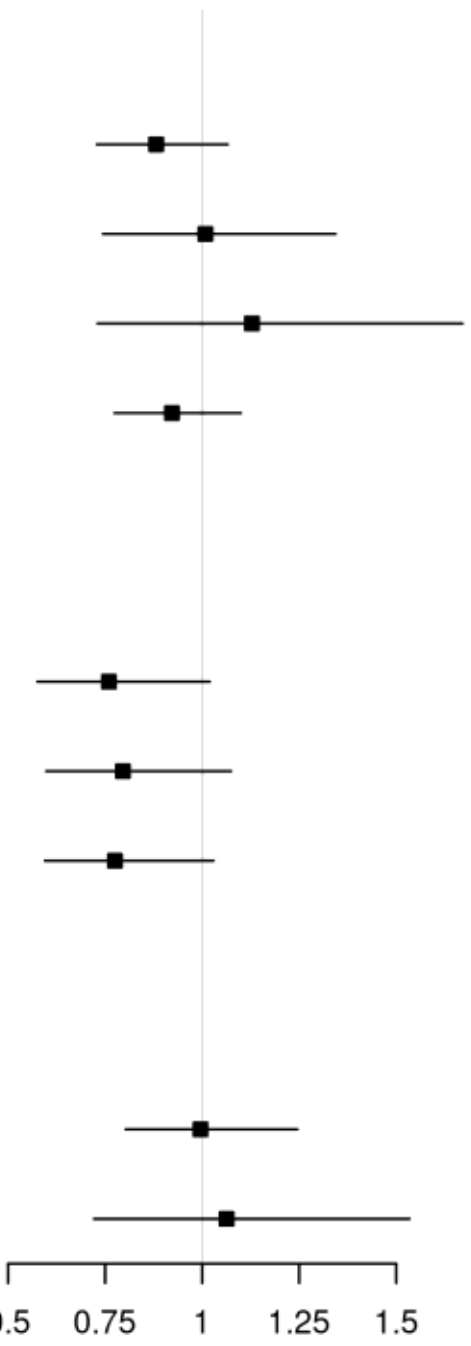

Adjusted for age, sex, diabetes, bmi, smoking status, alcohol use and 10 PCs
