# Supplementary material for: Genetically determined ABO blood group, secretor status, Lewis antigens, and panNEN risk
Source: Endocr Relat Cancer. 2026 Sep 18;33(9):e260286. doi: 10.1530/ERC-26-0286 (PMC13615866; doi:10.1530/ERC-26-0286)
Supplement: Supplementary file 6 [file ERC-26-0286_supplementary_tables.pdf]

Supplementary Table 1. Sample selection based on post-sequencing quality control checks.

	panNEN cases	Biobank Controls
Starting sample size	1069	52,913
**Reason for exclusion**		
Sex discordance	2	42
Exome capture 20x <80%	2	9
Contamination >5%	0	19
Duplicates	0	39
Related individuals	6	4589
Controls with prior history of any cancer type	0	15,197
**Final analytic sample**	1059	33,018

Supplementary Table 2. Derivation of ABO blood group and blood group-related glycosylation markers

ABO allele	rs505922	Haplotypes/Genotypes<br>rs8176693<br>(proxy for rs8176746)	rs574347
O	T	C	T/C
A	C	C	T
B	C/T	T	T/C
**Secretor status**	**rs601338**		
Secretor	GG or AG		
Non-secretor	AA		
**Lewis antigens**	**rs812936**	**rs28362459**	**rs3894326**
Positive (normal)	AA	AA	AA
Positive (semi-normal)	AA	AA	TA
	AA	AC	TA
	AA	AC	AA
	AA	CC	AA
	AG	AA	AA
Negative (no activity)	AA	AC	TT
	AA	CC	TA
	AA	CC	TT
	AG	AC	AA
	AG	AC	TA
	AG	CC	AA
	AG	CC	TA
	GG	AA	AA
	GG	AC	AA
	GG	AC	TA

Adapted from Kim et al., (PMID: 37342060).

Supplementary Table 3. Power calculations[a] for the association between ABO blood group (non-O vs O) and risk of panNENs, based on 1,059 cases and 33,018 controls, assuming effect sizes reported for PDAC (ORs, 1.28–1.50). Assume test significance level alpha=0.05, 2-sided test, 610 (57.6%) cases with non-O ABO blood group.

Controls, Observed	20950	21000	21500	21750	21900	22100	22150
Controls, Proportion	0.635	0.636	0.651	0.659	0.663	0.669	0.671
Odds ratio	1.281	1.286	1.373	1.423	1.448	1.488	1.501
**Power (%)**	**97**	**97**	**99**	**99**	**>99**	**>99**	**>99**

[a]Power was calculated using NQuery Advisor v70 power calculation software.

Abbreviations: panNEN, pancreatic neuroendocrine neoplasms; PDAC, pancreatic ductal adenocarcinoma; OR, odds ratios
